# Dynamin 2, cell trafficking, and the triple-negative paradox

**DOI:** 10.18632/oncotarget.26778

**Published:** 2019-03-22

**Authors:** Sophia B. Chernikova, John C. Game, J. Martin Brown

**Affiliations:** Stanford University, Department of Neurosurgery, Stanford, CA, USA

**Keywords:** triple-negative, dynamin 2, breast cancer, homologous recombination

Triple-negative breast cancer (TNBC) is the most aggressive subtype of breast cancer (BC). It is so named because it lacks expression of estrogen/progesterone receptors and does not overexpress the human epidermal growth factor receptor 2 (HER2). While only 15–20% of all BC cases are classified as TNBCs, they account for over 50% of BC mortality. TNBCs include the BRCA1/2- defective BCs and largely overlap with the basal-like BC group. As of today, no targeted therapies are available for these BC subtypes and despite advances in cytotoxic therapies they still carry a grim prognosis, with relapses typically occurring as soon as 3 years after treatment [1].

**Figure 1:**
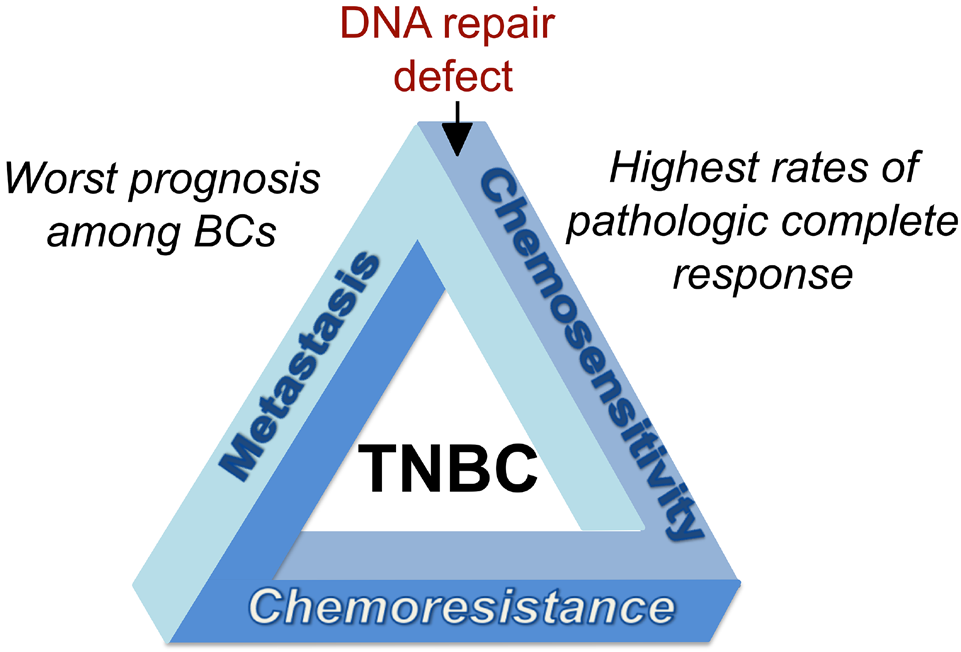
Triple-negative paradox of breast cancer Triple-negative breast cancer (TNBC, so called because it lacks expression of estrogen and progesterone receptors and does not overexpress the human epidermal growth factor receptor2 (HER2)) is the most sensitive to chemotherapy among all BC subtypes because of underlying defects in DNA repair. Despite the high responsiveness to chemotherapy among BCs, TNBCs carry the worst prognosis, with high resistance to chemotherapy at later stages and the lowest disease-free and overall survival. TNBCs are prone to local and metastatic relapse typically occurring within a short time (3–5 years) after chemotherapy.

TNBCs trace their origin to the genomic instability (GI) associated with defects in DNA repair (notably homology-directed repair (HDR)) and thus, compared to other BCs show increased rates of pathologic complete response following chemotherapy [1]. Yet, despite this initial high responsiveness to chemotherapy, TNBCs regain HDR capability and develop chemotherapy resistance in late-stage cancer, resulting in a much poorer prognosis compared to non-TNBCs. Known as the triple-negative paradox (Figure 1) [1] this phenomenon highlights the need to dissect biological pathways involved in the chemotherapy response and the evolution of resistance in these tumors.

We have recently shown that the efficiency of HDR depends on dynamin 2 (DNM2), a large GTPase best known for its role in intracellular molecular trafficking [2]. DNM2 inhibition hampered all aspects of HDR and increased sensitivity to a DNA cross-linking chemotherapy in various cell types. Intriguingly, although DNM2 inhibition increased sensitivity to chemotherapy in both non-TNBC and TNBC cells, tumor levels of DNM2 only affected survival of patients with estrogen receptor (ER)-negative BCs, including TNBCs, and not with ER-positive BCs. In particular, elevated expression of *DNM2* was associated with lower relapse-free survival and very short times (3–5 years) to relapse after chemotherapy in TNBC. Below we provide a mechanism that explains how increased DNM2 expression may shape resistance in later stages of the disease and why it is so uniquely important for the outcome in TNBC.

High genomic instability (GI) is a characteristic feature of TNBCs. One mechanism often employed by TNBCs to counteract GI is overexpression of RAD51, a protein central to HDR [3]. As a DNA repair protein, RAD51 is mostly nuclear, however a significant fraction of RAD51 is found in the cytoplasm. We have shown that cytoplasmic RAD51 is confined to microtubule-associated vesicles, which enable trafficking between nucleus and cytoplasm [2]. Up-regulation of RAD51 has been reported to rescue HDR defects induced by knockout of BRCA and some other HDR proteins [3]. We predicted that overexpression of *RAD51* might similarly reverse the HDR defects induced by DNM2 deficiency. However, although we successfully rescued the DNM2 inhibition-induced HDR phenotypes in all other cells, this was not the case for cells derived from advanced TNBCs, which remained strikingly dependent on DNM2 function for their survival after treatment with chemotherapy. We concluded that in the absence of BRCA and other proteins that control recruitment of RAD51 to the sites of DNA damage and/or stalled replication forks, RAD51 trafficking to the nucleus and subsequent cell resistance to chemotherapy was largely dependent on DNM2. This reasoning is supported by observations of aberrant cytoplasmic-to-nuclear ratios of RAD51 in late-stage TNBCs, suggesting the importance of RAD51 trafficking between nucleus and cytoplasm for the aggressiveness of TNBC [4].

Recent single-cell sequencing of longitudinal TNBC samples [5] has shown that resistance in TNBCs arises due to selection and expansion of rare pre-existing clones, rather than through induction of new mutations. Clones with increased DNM2 would have a selective advantage in response to treatment and thus take over the entire population within the TNBC tumors. Given that DNM2 also drives cell migration and invasion [6], the revamped population emerging after that evolutionary makeover would not only be resistant to chemotherapy but would also become highly metastatic, explaining the aggressive clinical behavior of TNBCs, known for their highest risk among all BCs for distant relapses and propensity to metastasize to leptomeninges.

**Figure 2:**
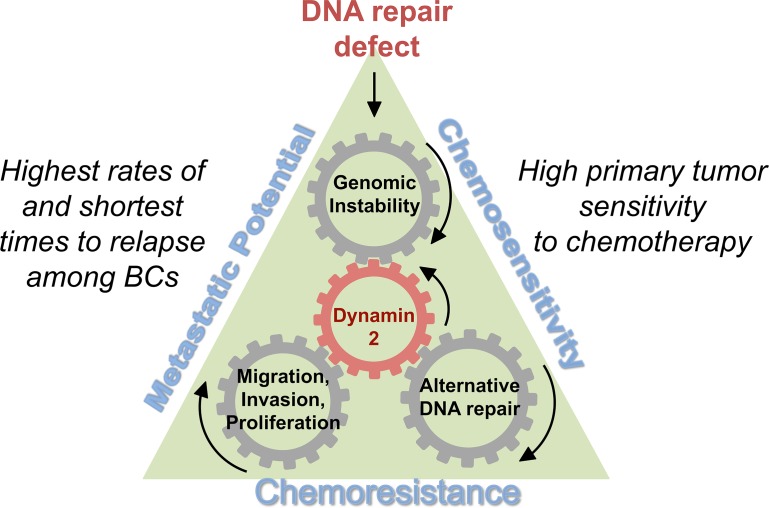
Increased DNM2 and associated intra-cell trafficking provide a possible solution to the triple-negative paradox DNA repair defects typical in TNBCs lead to increased genomic instability and account for the initial sensitivity to chemotherapy. To adjust to high levels of genomic instability some cells elevate Dynamin 2 (DNM2)-dependent protein trafficking. DNM2-dependent protein trafficking increases the efficiency of HDR, allowing TNBC cells to develop resistance to chemotherapy. These resistant cells have a selection advantage and may dominate the tumor population at later stages. DNM2 is also known to drive cell migration and invasion, therefore DNM2-overexpressing clones also become more metastatic.

In summary, increased DNM2 and associated intra-cell trafficking explain how DNA repair-deficient cells could acquire both resistance to chemotherapy and mobility, thus providing one possible solution to the triple-negative paradox of BC (Figure 2). The implications from our study are promising, but several questions remain. For example, does DNM2 stand alone in its ability to drive both treatment resistance and cell motility? Recent research shows that intracellular protein trafficking itself emerges as a common mechanism that impacts sensitivity to genotoxic agents and contributes to metastatic spread of cancer [7]. Elucidating the other players/sub-pathways and how they contribute to TNBC may move us a step closer towards developing comprehensive targeted therapies for TNBC and other hormone-negative BCs.
